# A Triad of Lys12, Lys41, Arg78 Spatial Domain, a Novel Identified Heparin Binding Site on Tat Protein, Facilitates Tat-Driven Cell Adhesion

**DOI:** 10.1371/journal.pone.0002662

**Published:** 2008-07-16

**Authors:** Jing Ai, Xianliang Xin, Mingyue Zheng, Shuai Wang, Shuying Peng, Jing Li, Limei Wang, Hualiang Jiang, Meiyu Geng

**Affiliations:** 1 Department of Pharmacology and Glycobiology, Marine Drug and Food Institute, Ocean University of China, Qingdao, People's Republic of China; 2 Division of Anti-tumor Pharmacology, State Key Laboratory of Drug Research, Shanghai Institute of Materia Medica, Chinese Academy of Sciences, Shanghai, People's Republic of China; 3 Drug Discovery and Design Center, State Key Laboratory of Drug Research, Shanghai Institute of Materia Medica, Chinese Academy of Sciences, Shanghai, People's Republic of China; 4 Laboratory of Mass Spectrometry, Departmant of Analytical Chemistry, Shanghai Institute of Materia Medica,Chinese Academy of Sciences, Shanghai, People's Republic of China; University of Helsinki, Finland

## Abstract

Tat protein, released by HIV-infected cells, has a battery of important biological effects leading to distinct AIDS-associated pathologies. Cell surface heparan sulfate protoglycans (HSPGs) have been accepted as endogenous Tat receptors, and the Tat basic domain has been identified as the heparin binding site. However, findings that deletion or substitution of the basic domain inhibits but does not completely eliminate Tat–heparin interactions suggest that the basic domain is not the sole Tat heparin binding site. In the current study, an approach integrating computational modeling, mutagenesis, biophysical and cell-based assays was used to elucidate a novel, high affinity heparin-binding site: a Lys12, Lys41, Arg78 (KKR) spatial domain. This domain was also found to facilitate Tat-driven β1 integrin activation, producing subsequent SLK cell adhesion in an HSPG-dependent manner, but was not involved in Tat internalization. The identification of this new heparin binding site may foster further insight into the nature of Tat-heparin interactions and subsequent biological functions, facilitating the rational design of new therapeutics against Tat-mediated pathological events.

## Introduction

The transactivating factor of the HIV-1 virus (Tat), released from human immunodeficiency virus type 1 (HIV-1) infected cells, has been pointed towards a variety of important biological functions related to the distinct AIDS-associated pathologies in AIDS patients, including neuropathies[1], and immune suppression[2], [3] and increased tumorigenesis in AIDS patients[3].

Tat is a polypeptide of 86–102 amino acids depending on the viral strain[4]. Amino acids 1–72 are endowed with full transactivating activity[5], present within a basic domain (amino acids 48–57) constituted by a stretch of repeated Arg and Lys residues critical for a number of biological functions, while amino acids 72–86 of the carboxy terminal region contain an Arg-Gly-Asp (RGD) motif responsible for binding to integrin receptors and subsequent cell adhesion[4], [6].

Tat-driven activities depend precisely on its interaction with cell surface heparan sulfate (HS) and heparin through their negatively charged sulfate groups[7]. The basic domain has long been recognized as the sole contributor mediating this interaction via its heparin binding properties[8], [9], [10], [11]. However, recent limited studies have challenged this notion with the demonstration that deletion or substitution of the basic domain really affects, but does not completely eliminate Tat-heparin interactions[9]. This led us to hypothesize that other heparin binding sites might exist.

Toward this end, we sought to identify novel Tat heparin binding sites using the molecular simulation for appreciable prediction, combined with the surface plasmon resonance (SPR)-based competitive inhibition assay and cell-based functional evaluation with the introduction of several relevant targeted mutant Tat proteins. Encouragingly, a triad of basic residues—Lys12, Lys41 and Arg78 (KKR)—that are spatially enclosed rather than sequentially oriented, was thus identified as a hereto unrecognized high-affinity heparin binding site which help facilitate Tat-driven β1 integrin activation and subsequent adhesion in an heparan sulfate protoglycan (HSPG)-dependent manner. Clearly, our findings will facilitate a crucial touch on a precise understanding of the comprehensive roles of Tat as well as aid in design of new therapeutic agents.

## Results

### Molecular simulation predicts that Lys12, Lys41, and Arg78 comprise a novel Tat heparin binding site

**Figure 1:**
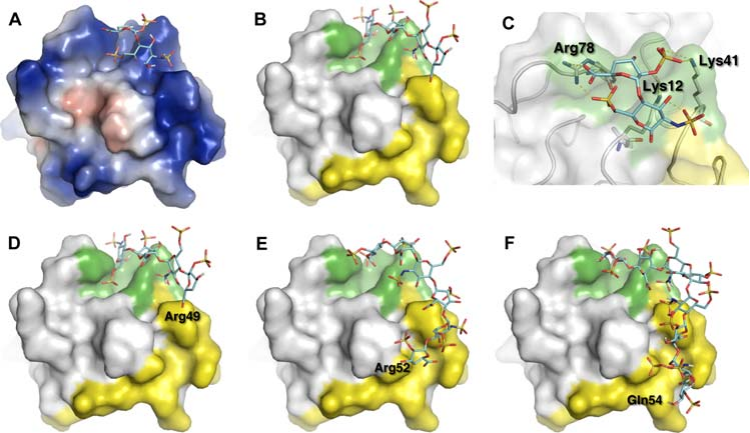
Computational docking model of HIV-Tat with heparin-derived fragments. Predicted interaction modes between HIV-Tat and heparin-derived fragments, including di- (A, B and C), tetra- (D), hexa- (E), and octa-saccharide (F) were indicated. Tat was represented in surface and oligosaccharides in stick. The Tat surface was colored by electrostatic potential in A. In B–F, it was colored in white, with the KKR region and basic domain highlighted in green and yellow, respectively. A close-up view of the binding interface between the di-saccharide and the KKR (Lys12, Lys41 and Arg78) region was shown in C, where the surface was set to transparent, the protein main chain was shown in ‘worm’ representation, and hydrogen-bonds are represented by yellow dotted lines.

Computational modeling was first employed with the aim of identifying the potential heparin binding sites. Here, the homology model of Tat protein (Tat-III) constructed on the basis of NMR data was utilized as the target structure, and several heparin oligosaccharides including di-, tetra-, hexa-, and octa-saccharides were selected as docking probes. In the disaccharide heparin model, heparin was noted to preferentially interact with a triad of basic residues (Lys12, Lys41 and Arg78 (KKR); Figure 1, highlighted in green). For better resolution, a closeup view of the binding interface was developed. In the zoomed view, It was evident that the 2-O-sulfate of the glucuronic acid ring was in close proximity to Lys41 in N_ζ_, which itself is engaged in two tight salt bridges (2.84 and 2.88 Å, respectively). N_ζ_ atom of Lys12, in a like manner, was involved in two hydrogen bonds, established with the 3-O (3.30 Å) and 2-O (2.74 Å) of the glucuronic acid ring. Another strong salt bridge (2.77 Å) was observed between the 5-carboxylate group and N_η_ of Arg78. The binding free energy of the disaccharide was further calculated as −8.84 kcal/mol, indicative of the nanomolar or even lower level of the binding affinity of Tat-III for heparin.

Of particular note, among the 20 distinct docking runs, though a few predicted poses were docked at the basic domain, it however generated much higher estimated binding free energies. These finding from an alternative view suggested that the KKR triad may yield a higher affinity for heparin than that of the basic domain.

Modeling of interactions with tetra-, hexa- and octa-saccharides revealed similar results; the positively charged KKR triad plays an important role in the molecular recognition of heparin probes producing a highly conserved interaction with Tat-III. It should be noted that the oligosaccharide chain begins to stretch toward the basic domain (yellow for comparison) as the probe length increases. As the length increases to a tetramer, a delicate interaction with residue Arg49 of the basic domain is established; the hexamer follows the same path and approaches residue Arg52; when the heparin oligosaccharide reaches octameric size, it enlists residue Gln54. Their binding free energies were calculated as −9.21, −11.53 and −12.30 kcal/mol, respectively. Heparin ideally, if long enough, is proposed to encompass both the KKR region and fully extend to the whole basic domain; and Arg49 might serve as a ‘knot/link’ between the two binding sites.

### Surface plasmon resonance confirms that the KKR domain is a spatially enclosed Tat heparin binding site

**Figure 2:**
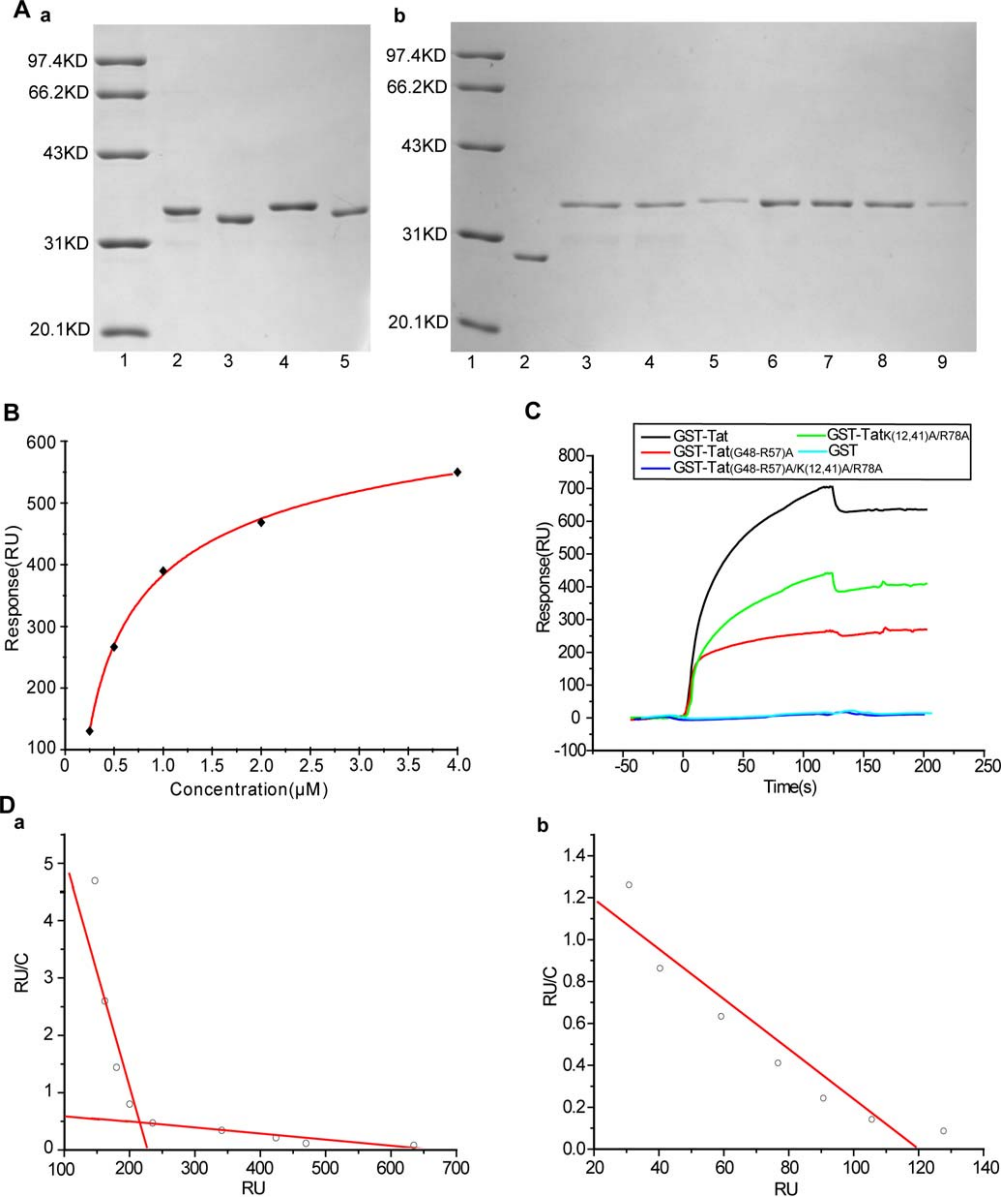
Identification of the Tat Lys12, Lys41, Arg78 (KKR) domain as a new heparin binding site. A, SDS-PAGE analysis of the purification of GST-Tat and series Tat mutants . a, 1, Marker; 2, GST-Tat; 3, GST-Tat_(G48-R57)A_; 4, GST-Tat_K(12,41)A/R78A_; 5, GST-Tat_(G48-R57)A/K(12,41)A/R78A_. b, 1, Marker; 2, GST; 3, GST-Tat_K12A_; 4, GST-Tat_K41A_; 5, GST-Tat_R78A_; 6, GST-Tat_K(12,41)A_; 7, GST-Tat_K12A/R78A_; 8, GST-Tat_K41A/R78A_; 9, GST-Tat_R78A/G79A/D80A_. B, Binding activities of GST-Tat to heparin immobilized on a CM5 sensor chip. Varying concentrations of GST-Tat (0.25, 0.5, 1, 2, 4 µM) were injected over the heparin-immobilized sensor chip surface, and binding of GST-Tat to heparin was recorded as RU. C, Interaction of GST-Tat and its mutants with biotinylated heparin immobilized on a CM5 sensor chip. GST-Tat or the appropriate mutants (1.5 µM) were injected over the heparin-immobilized sensor chip. The binding RU was recorded as binding potency. D, Scatchard plot analysis of GST-Tat and GST-Tat_(G48-R57)A_ with immobilized heparin. Series concentrations of GST-Tat or GST-Tat_(G48-R57)A_ was injected over the heparin-immobilized sensor chip surface. The binding potency/concentration (RU/C) was plotted versus RU. (a) and (b) stand for Scatchard plots for GST-Tat, GST-Tat_(G48-R57)A_ respectively.

To characterize the Tat KKR motif engaged with heparin, a series of Tat mutants were expressed in *E. coli* as GST fusion proteins, purified from the bacterial lysates by glutathione-agarose affinity chromatography and checked by SDS-PAGE (Figure 2A). The identification of mutant products was further carried out by MS-MS spectral analysis (Supplemental Text S1 and Figure S1, S2, S3, S4). In addition, the similarity of secondary structure of the mutated products to the native Tat protein was predicted using a position-specific scoring method [12](data not shown).

First, the EC_50_ binding capacity was evaluated. For this, a series of GST-Tat solutions with a range of concentrations (0.25, 0.5, 1, 2, and 4 µM) were injected over a heparin-immobilized sensor chip surface. RUmax values were generated using BIAcore evaluation 3.1 software, yielding an EC_50_ value of 1.5 µM (Figure 2B); this value was used as a reference for subsequent experiments.

We next investigated whether KKR is a heparin binding site, as predicted. A series of comparative studies were performed using all relevant mutants. We found that GST-Tat binding to heparin involves multi-phasic binding (Figure 2D)[13], indicating the existence of more than one heparin binding site. Notably, mutation of the basic domain still produced monophasic binding, further supporting the notion that another binding site for heparin exists in addition to the basic domain. Accordingly, the mutation of Lys12, Lys41, Arg78 with alanine [GST-Tat_K(12,41)A/R78A_] displayed a markedly decreased binding potency for heparin when compared to wild-type GST-Tat (Figure 2C). Moreover, double-mutant GST-Tat_(G48-R57)A/K(12,41)A/R78A_ led to the complete abolishment of Tat-heparin interactions. These findings substantiate our hypothesis that Tat indeed possesses another heparin binding site, identified herein as the KKR region.

We next clarified the contribution of the three basic residues. Single, double or triple mutations of the KKR region were constructed and their binding affinities for immobilized heparin were recorded and compared using the SPR assay. K_D_ values were calculated using BIAcore evaluation 3.1 software. Mutation of a single residue (GST-Tat_K12A_, GST-Tat_K41A_, or GST-Tat_R78A_) led to only a partial loss of heparin binding affinity (K_D_ 0.275 nM, 0.155 nM or 0.0241 nM, respectively), with the Arg78 mutation (GST-Tat_R78A_) influencing binding affinity the least. Moreover, mutation of any two KKR residues (GST-Tat_K(12,41)A_, GST-Tat_K12A/R78A_, GST-Tat_K41A/R78A_) led to a significant loss of binding affinity with correspondingly decreased K_D_ values (1.42 nM, 0.249 nM and 0.154 nM), with Lys12, Lys41 mutation (GST-Tat_K(12,41)A_) affecting binding the most. Most significantly, concurrent mutation of all three residues produced a dramatic drop in heparin binding affinity, with a resultant K_D_ value of 32.6 nM. This series of experiments collectively indicated that Lys12, Lys41 and Arg78 cooperate to profoundly impact heparin-binding (Table 1).

**Table 1:** The binding affinity parameters of GST-Tat and its mutants to heparin.

Analyte	K_A_(1/M)	K_D_ (M)
GST-Tat	8.56E+11	1.17E−12
GST-Tat_(G48-R57)A_	1.88E+11	5.31E−12
GST-Tat_K (12, 41)A/R78A_	3.07E+07	3.26E−08
GST-Tat_K12A_	3.64E+09	2.75E−10
GST- Tat_K41A_	6.45E+09	1.55E−10
GST- Tat_R78A_	4.15E+10	2.41E−11
GST- Tat_K(12, 41)A_	7.05E+08	1.42E−09
GST-Tat_K12A/R78A_	4.02E+09	2.49E−10
GST- Tat_K41A/R78A_	6.51E+09	1.54E−10

K_A_: equilibrium association constant; K_D_: equilibrium dissociation constant. Data shown are typical from three independent experiments with similar results.

### The KKR spatial triad binds specifically to heparin in a high affinity manner

**Figure 3:**
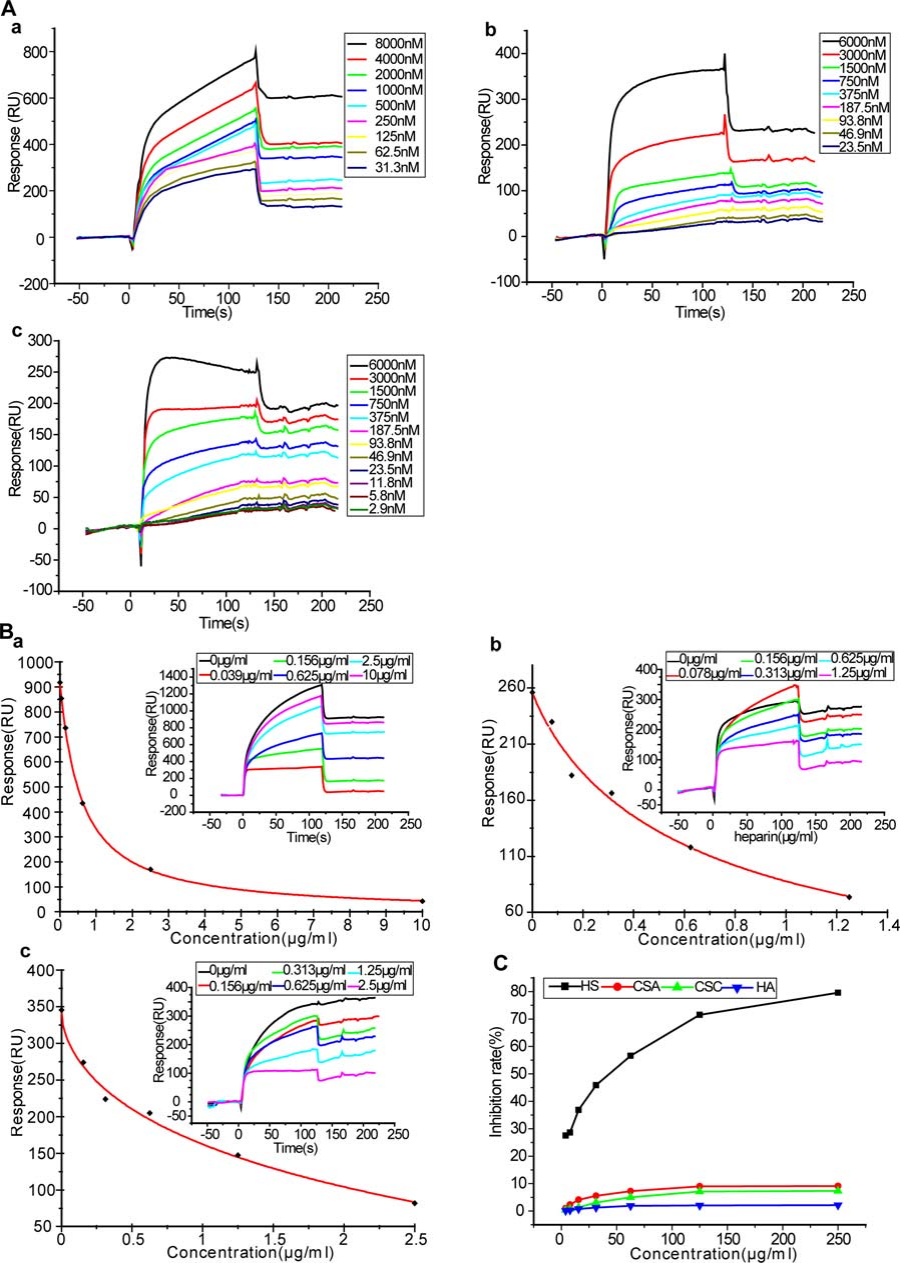
Characterization of the intrinsic properties of the Tat KKR heparin binding domain. A, Sensorgrams of GST-Tat and its mutants with immobilized heparin. Serial concentrations of GST-Tat and its mutants were injected over the heparin-immobilized sensor chip surface. The real time binding were recorded as response (RU) versus time. (a), (b) and (c) represent sensorgrams for GST-Tat, GST-Tat_(G48-R57)A_, and GST-Tat_K (12,41)A/R78A_ respectively. B, Heparin competitively inhibits binding of GST-Tat and its mutants with immobilized heparin. GST-Tat (a), GST-Tat_(G48-R57)A_ (b) and GST-Tat_K (12,41)A/R78A_ (c) (1.5 µM) alone or in the presence of serial concentrations of heparin were injected over an heparin immobilized chip surface, respectively. The responses (in RU) were plotted versus the concentration of heparin. Insets, overlay of sensograms showing the binding of GST-Tat and its mutants to immobilized heparin in the presence of increasing concentrations of heparin. C, The inhibitory action of different glycosaminoglycans on GST-Tat_(G48-R57)A_-heparin interaction. GST-Tat_(G48-R57)A_ (1.5 µM) was preincubated with increasing concentrations of free HS, CSA, CSC, and HA (7.8, 15.6, 31.3, 62.5, 125, 250 µg/ml), respectively, and injected over a biotinylated heparin surface.

We next sought to delineate the nature of KKR-associated heparin binding in an attempt to define the intrinsic properties of this newly discovered binding site. We studied binding affinity (K_D_), a key feature of the binding mode. A series of concentrations of GST-Tat, GST-Tat_(G48-R57)A_ and GST-Tat_K (12,41)A/R78A_ were injected over the heparin-immobilized sensor chip surface (Figure 3A). We found that GST-Tat is capable of binding to heparin (K_D_ = 1.17 pM) (Table 1). Mutation of the basic domain (leaving the KKR domain intact and thus solely responsible for the strength of the Tat KKR-heparin interaction) led to a decrease in binding affinity (K_D_ = 5.31 pM). This value represents nearly a five-fold reduction in affinity as compared to wild-type GST-Tat. Mutation of the KKR region, however, caused a remarkable decrease in GST-Tat-heparin binding (K_D_ = 32.6 nM), representing the K_D_ value of the basic domain in isolation. This K_D_ represents a 10,000 fold decrease in binding affinity compared with that of wild-type GST-Tat (Table 1). Based on this comparison, we conclude that the KKR spatial region dominates Tat-heparin binding in comparison to the basic domain.

We next conducted a series of experiments evaluating heparin-mediated competitive inhibition of Tat-heparin binding for further confirmation. Wild-type GST-Tat or the appropriate Tat mutants (1.5 µM) was injected over a heparin-immobilized chip in the presence of varying concentrations of heparin as a competitive inhibitor. Free heparin was able to inhibit the binding of either GST-Tat or the corresponding mutant to immobilized heparin in a dose-dependent manner (Figure 3B). Notably, mutation of the basic domain (GST-Tat_(G48-R57)A_) produced an IC_50_ value (0.659 µg/ml) comparable to that of GST-Tat (0.717 µg/ml), while mutation of the KKR region (GST-Tat_K (12,41)A/R78A_) yielded an IC_50_ value of 1.574 µg/ml, almost twice that of GST-Tat. These data further support the notion that the KKR spatial domain contributes dramatically to heparin binding, and represents an extremely high-affinity heparin binding site.

We then characterized KKR binding specificity, another important component of the binding mode, via introduction of a series of soluble glycosaminoglycans (GAGs) using competitive inhibition assay. Both heparin and HS were able to compete for KKR binding to heparin, yielding IC_50_ values of 0.717 µg/ml and 20.677 µg/ml respectively. By contrast, other GAGs including CSA, CSC and HA, even at concentrations up to 250 µg/ml, showed little or no inhibition of this interaction (Figure 3C). These findings support the high specificity of the KKR region for heparin.

Thus, the newly identified KKR region, distinct from the well-recognized linearly contiguous basic domain, serves as both an extremely high-affinity and highly specific binding motif for heparin.

### The KKR spatial region is not involved in Tat-driven internalization

**Figure 4:**
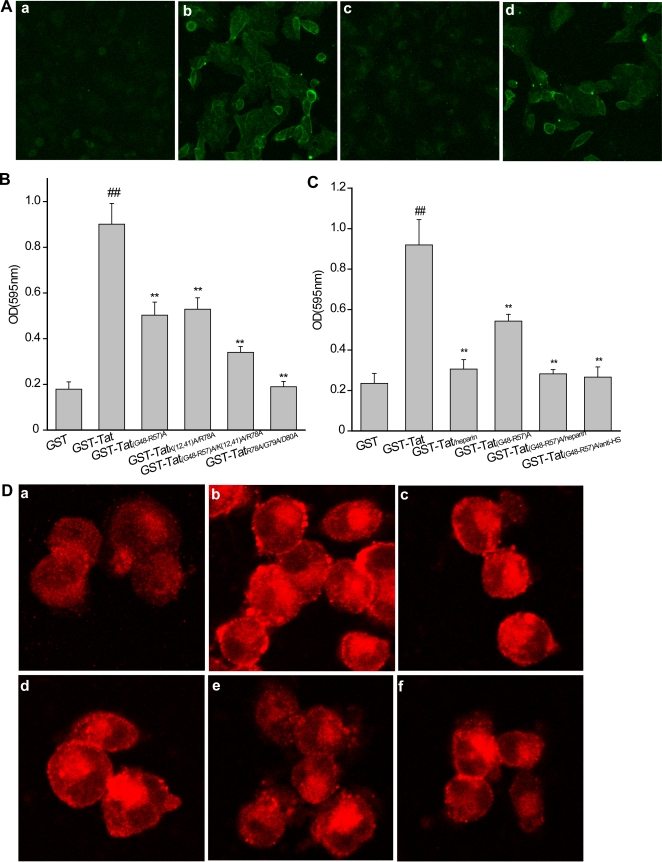
Effects of the KKR domain on Tat-mediated internalization and adhesion in SLK cells. A, Internalization of extracellular Tat fusion protein and its mutant derivatives in SLK cells. Cells were treated with GST-Tat or its mutants (1.6 µg/ml). After 24 h, cells were extensively washed, fixed, and internalized fusion protein was stained with monoclonal anti-GST antibody [1∶100], followed by FITC-conjugated goat anti-rabbit IgG secondary antibody [1∶500] (green) and detected with immunofluorescence confocal microscopy. (a)GST, (b)GST-Tat, (c)GST-Tat_(G48-R57)A_, (d) GST-Tat_K (12,41)A/R78A_. B, Effect of immobilized Tat and its mutants on SLK cell adhesion (*x̅*±*s*, n = 3), ##p<0.01 vs GST; **p<0.01 vs. GST-Tat. C, Effect of soluble heparin and blockade of cell surface heparan sulfate on GST-Tat_(G48-R57)A_ adhesion in SLK cells (*x̅*±*s*, n = 3), ##p<0.01 vs GST; **p<0.01 vs. GST-Tat_(G48-R57)A_. D, Effect of KKR domain mutation on β1-integrin clustering in SLK cells. Activated β1 integrin was detected using monoclonal antibody 12G10, and probed using Cy3-conjugated sheep anti-mouse IgG (red). (a) GST, (b) GST-Tat, (c) GST-Tat_(G48-R57)A_, (d) GST-Tat_K (12,41)A/R78A_ (e) GST-Tat_(G48-R57)A/K(12,41)A/R78A_, (f) GST-Tat_(G48-R57)A_/heparin.

It is well-known that Tat-regulated function mostly precisely depends on the association of Tat with cell surface heparin sulfate/heparin via the negatively charged sulfate group. Of note, Tat-driven internalization indispensably counts on Tat's heparin-binding properties[10]. We thus examined whether the KKR region is involved in this critical event. For this purpose, SLK cells were preferentially adopted. As shown in Figure 4A, GST-Tat is capable of internalizing into SLK cells, reaching maximal intracellular concentration within 24 h, and consistently, the mutation of the basic domain (GST-Tat_(G48-R57)A_) is expected to cause a complete loss in Tat internalization[10], again indicating the dominance of the basic domain in this setting. The substitution of KKR region with alanine (GST-Tat_K(12,41)A/R78A_), however, failed to affect internalization at all. This helps exclude the involvement of the KKR spatial domain in Tat protein internalization.

### The KKR spatial region contributes to Tat-mediated SLK cell adhesion via β1 integrin activation in a cell surface HSPG-dependent manner

#### The KKR spatial region facilitates Tat-mediated SLK cell adhesion

In addition to the Tat-driven internalization, Tat also mimics the effect of extracellular matrix molecules and favors cell adhesion via the amino acid sequence RGD[4], [6]. We thus reasoned that KKR might involve the Tat-mediated RGD-executed cell adhesion. For this, we again selected SLK cells as a surrogate to examine the impact of KKR on cell adhesion. Replacement of KKR with alanine (GST-Tat_K(12,41)A/R78A_) induced a partial but incomplete abolishment of SLK cell adhesion compared with that of GST-Tat (Figure 4B), suggesting this new heparin binding site is at least partially involved in Tat-mediated cell adhesion. In comparison, substituting RGD with alanine (GST-Tat_R78A/G79A/D80A_) resulted in a complete defect in cell adhesion. These findings together underscore the predominant role of RGD on one hand and meanwhile revealing a complementary action of KKR in Tat-driven cellular adhesion. Furthermore, mutation of the basic domain (GST-Tat_(G48-R57)A_) generated a similar profile, as did double mutation of both KKR and the basic domain (GST-Tat_(G48-R57)A/K(12,41)A/R78A_) which yields a more potent reduction in cell adhesion compared with KKR-mutated Tat. These findings further support the concept that the KKR spatial triad is an essential, but incomplete, contributor to Tat-mediated cell adhesion.

#### The KKR region triggers β1 integrin activation in SLK cells

Since Tat promotes SLK cell adhesion through binding α5β1 integrins[6], [14], we next evaluated whether the KKR drives this activation. Activation of β1 integrin was recorded in GST-Tat-treated SLK cells, as evident from the typical dot-like staining observed in peripheral ruffles and cell edges (Figure 4D)[15], [16], [17]. In contrast, an appreciable loss of dot-like staining was observed in SLK cells after treatment with GST-Tat_K(12,41)A/R78A_. GST-Tat_(G48-R57)A_ treatment resulted in conserved diffuse β1 integrin staining, while GST-Tat_(G48-R57)A/K(12,41)A/R78A_ produced less β1 integrin activation. However, the activated β1 integrin induced by GST-Tat_R78A/G79A/D80A_ couldn't be detected due to its disability on cell adhesion (data not shown). All these findings are concordant with the results obtained in the cell adhesion assay, which is the consequence of β1 integrin activation.

#### The KKR region executes its function in a cell surface HSPG-dependent manner

To address whether cell surface HSPG acts as a chaperone for KKR-facilitated cell adhesion events in the context of its heparin binding properties, we performed a series of competitive inhibition assays designed to test its effects on cell adhesion. This was accomplished either by introducing soluble heparin or a specific monoclonal antibody raised against cell surface HS (MAB2040). GST-Tat-induced SLK cell adhesion was inhibited by heparin at concentrations of 200 µg/ml (Figure 4C). Furthermore, heparin, at the same concentration, competitively suppressed SLK cell adhesion induced by KKR-dominant but basic domain-substituted Tat [GST-Tat_(G48-R57)A_]. Consistently, MAB2040 also significantly blocked SLK cell adhesion facilitated by KKR-dominant but basic domain-substituted Tat [GST-Tat_(G48-R57)A_], indicating that KKR-mediated adhesion is heparin-dependent.

We subsequently examined the effects of heparin on KKR-facilitated β1 integrin activation. Here, pretreatment with heparin at a concentration of 1 mg/ml resulted in a competitive loss of β1 integrin staining induced by KKR-dominant but basic domain-substituted Tat [GST-Tat_(G48-R57)A_] (Figure 4D). Together, these results clearly suggest that the KKR region executes its biological function in a cell surface HSPG-dependent manner secondary to its heparin binding properties.

## Discussion

Rusnati et al.[9] previously recognized that the neutralization of six arginine residues within the basic domain was not sufficient to abolish the heparin binding capacity of Tat. They suggested that the remaining lysine residues in the basic region and/or other basic amino acids scattered on the surface of the Tat protein contributed to the residual heparin binding capacity they observed. Since that time, however, limited studies have touched this inconsistency in existing models of Tat-heparin interactions. With the availability of NMR-based Tat structure models together with progress that has been made in molecular simulations as well as the SPR technique, we were able to challenge the existing dogma and provide a direct evidence that it is a triad KKR region that functions as the crucial determinant of Tat-heparin interaction in addition to the basic domain.

In the present study, a homology model of Tat-III constructed using its NMR structure docked with heparin oligos probes provided a promising candidate motif for heparin binding in addition to the well-recognized basic domain. Further analysis of the free energy of heparin-Tat-III interactions and detailed assessment of their atomic associations, coupled with flexible molecular docking schemes substantially predicted the confluence of a triad of basic residues Lys12, Lys41, Arg78 (designated as KKR triad) into a previously unrecognized binding site for heparin.

The SPR biosensor assay is an accurate and powerful method of dig the molecule-molecule interactions[18], [19], [20], while mutation studies provide a wealth of information about biological functions. By combining these two approaches, we successfully defined the intrinsic properties of the KKR domain and characterized its functional importance. Using the SPR assay, a multiphasic binding mode between Tat and heparin was observed. With mutation of the basic domain, a monophasic binding series became evident. A complete loss of binding capacity for heparin was then noted with double mutation of both KKR and the basic domain. These findings collectively support the idea that the Tat protein offers two binding sites for heparin: the well-recognized basic domain, and the newly identified KKR region. This model is congruent with our simulation-based predictions. Notably, the observation that mutants with either a single or a double substitution of KKR residues does not dramatically affect heparin binding affinity, while a triple mutation significantly did, highlights the importance of all residues in this triad for functionality.

Delineation of the intrinsic properties of the newly identified binding site is important since it assists in unraveling the nature of the domain's interaction with heparin. By kinetics analysis, the dissociation constant (K_D_ value) was calculated as 5.31 pM, which represents an extremely high-affinity binding interaction (∼10,000 times that of the basic domain). The disparity in binding affinity between the KKR and the basic domain, in principle, should allow heparin to preferentially occupy KKR. *In silico* simulations clearly demonstrate that heparin can specifically recognize the KKR region when the disaccharide probe was used. As the length of the oligosaccharide increases, the oligomer stretches toward and along the basic domain: it begins to form interactions with Arg49 when the tetramer probe was used, approaches Arg52 as the probe size increases to 6 sugar unit, and reaches Gln54 as the sugar length is 8. Ideally, heparin at its typical length would simultaneously bind to both KKR and the whole basic domain, with residue Arg49 serving as the ‘knot/link’ between the two regions.

Another important feature shaped by the KKR domain is its specificity for heparin, as is evident from the observation that only heparin/HS but not other GAGs including CSA, CSC and HA were able to produce a dose-response-based competitive inhibitory effect on Tat-heparin interactions. Because sugar-protein interactions are predominately dependent on saccharide composition and the extent and distribution of sulfation of the sugar backbone[21], [22], the high affinity of Tat for heparin may be a product of both its defined sulfate groups and specific saccharide composition. However, though heparin and HS are comprised of same saccharide composition, HS still displays differences from heparin in binding affinity for KKR triad, which clearly points to the importance of the extent of sulfation of sugar chains in specificity of sugar-protein recognition.

Current views hold that heparin binding sites are commonly recognized on the external surface of proteins and correspond to shallow pockets of positive charge that are often linearly contiguous or, alternatively, may be distant in sequence yet closely spatially orientated when the protein assumes its native tertiary structure[23], [24], [25]. Unlike the linearly contiguous sequence of the basic domain, the KKR residues, although distant in sequence, are brought into close spatial proximity via Tat conformational folding. This intrinsic difference might endow the KKR with a contribution to Tat function distinct from that of the basic domain. Indeed, the fact that the KKR is only capable of facilitating Tat-driven activation of β1 integrin and subsequent cell adhesion (similar to the basic domain), but fails to produce intracellular internalization (unlike the basic domain) favors such a notion. Of course, the delicate nature of heparin-KKR interactions will require further elucidation.

Regarding integrin activation and subsequent adhesion, one important issue merits further addressed: the KKR triad matches quite well in a similar pattern with RGD-driven events that depend on its engagement with cell surface HS[4]. However, a complete loss of integrin activation and subsequent cell adhesion via the replacement of the RGD region, but only a partial loss observed with KKR substitution, convincingly support the concept that the KKR is an essential but not dominant component in mediating cell adhesion events as RGD does. This triad, alternatively resembling the basic domain, cooperates with the RGD motif in integrin activation and subsequent adhesion.

In sum, we herein show for the first time that a triad of Lys12, Lys41, Arg78 domain is a novel, high-affinity, spatially enclosed heparin binding site on Tat, which we have further defined as important in facilitating Tat-driven integrin activation and subsequent cell adhesion in an HSPG-dependent manner. Of note, this newly identified heparin binding site functions similarly to the basic domain as a participator in Tat-triggered cell adhesion on one hand, but behaves quite differently from the basic domain in the internalization process. Particularly, the findings that in addition to the commonly accepted basic domain, the KKR region is an extremely high affinity heparin binding site appeal a careful consideration when interpreting Tat-driven adhesion events. Further characterization of functional synergy of KKR-mediated Tat-heparin interactions will add a crucial component of understanding AIDS-associated pathological in particular and Tat-driven battery of biological events in general. Also, challenging efforts towards the interest in the KKR-targeted rational designing for novel therapeutics are further needed.

## Materials and Methods

### Reagents

Heparin, HS, chondroitin sulfates A (CSA) and C (CSC), hyaluronic acid (HA) and Cy3-conjugated sheep anti-mouse IgG were obtained from Sigma (St. Louis, MO). Anti-GST antibody (sc-459) was purchased from Santa Cruz Biotechnology, Inc. (Santa Cruz, CA). Monoclonal antibodies to activated-integrin β1 (12G10) and HS (MAB2040) were obtained from Chemicon International, Inc. (Temecula, CA). FITC-conjugated goat anti-rabbit IgG was purchased from Jackson Immunoresearch Laboratory Inc. (West Grove, PA).

### Cell culture

SLK cells (derived from an endothelial-origin Kaposi's sarcoma) were kindly provided by the NIH AIDS Research division. The cells were maintained in RPMI1640 medium supplemented with 10% heat-inactivated fetal calf serum (FCS, Gibco Gaithhersburg, MD), 2 mM L-glutamine, 100 IU/ml penicillin, and 100 µg/ml streptomycin at 37°C in a humidified incubator with 5% CO_2_.

### Computational docking modeling

The amino acid sequence of human T-lymphotropic virus 3 (Tat-III protein) was retrieved from the appropriate NCBI Pubmed entry (http://www.ncbi.nlm.nih.gov/) (GI: 328765) for phylogenetic analyses. A BLASTP (http://www.ncbi.nlm.nih.gov/blast) search of the sequence revealed a highly homologous protein, Tat-I (83/86 aa, 96%identity), of which 11 NMR solution structures have been determined (PDB entry 1JFW)[26]. Therefore, the structure of Tat-I was used as the template to create a 3D model of Tat-III using the MODELLER program implemented in INSIGHT II(Insight II. UserGuide, MSI Inc., San Diego, USA, 2000) [27]. Ten models were generated, and these 3D structures were optimized with a conjugate gradient minimization scheme followed by a restrained simulated annealing molecular dynamics simulation. The model with the lowest value of the objective function was selected as the most representative Tat-III model for further study. For heparin, initial structural models of the tetrasaccharide and octasaccharide analogues were also built in INSIGHT II based on its NMR solution structure (PDB entry 1HPN)[28].

Structure-based analysis of our homology model of Tat-III was performed in order to search for novel heparin binding sites by using the advanced docking program AUTODOCK4.0[29], [30], [31]. The docking operation algorithm was performed in a consistent manner. In the first step, a large grid box encompassing the entire Tat-III structure, with 120×120×120 points, was generated to take all possible heparin binding sites into account. The spacing parameter was set to 0.375 Å and affinity and electrostatic potential maps were calculated for each type of atom present in the heparin structure. Then, the Lamarckian genetic algorithm (LGA) was applied to account for protein-ligand interactions using a newly revised scoring function that includes the terms vdw (Van der Waals), hydrogen bond, desolvation energy, torsional free energy and unbound system energy[29]. The step size was set to 0.2 Å for translation and 5° for orientation and torsion. The number of generations, energy evaluations, and docking runs were set to 500,000, 2,500,000 and 20, respectively. Finally, the evaluation with the lowest binding energy was used to analyze ligand pose and the interaction model of heparin/Tat-III was produced using the LIGPLOT program based on the docked complex structure[32].

### Preparation of Tat and its mutants

Plasmid pGEX-2T (Amersham Pharmacia, Uppsala, Sweden) and pGST-Tat were kindly provided by Profs. M Giacca and M Prestai (International Centre for Genetic Engineering and Biotechnology, Trieste, Italy). Tat mutants were constructed using site-directed mutagenesis, yielding series of GST-Tat mutants (Table 2).

**Table 2:** Summary of mutated amino acid of series GST-Tat mutants.

Mutants name	Amino acids change
GST-Tat_(G48-R57)A_	amino acids 48–57( basic domain)→Ala
GST-Tat_K(12,41)A/R78A_	Lys12, Lys41, Arg78→Ala
GST-Tat_K12A_	Lys12→Ala
GST-Tat_K41A_	Lys41→Ala
GST-Tat_R78A_	Arg78→Ala
GST-Tat_K(12,41)A_	Lys12, Lys41→Ala
GST-Tat_K12A/R78A_	Lys12, Arg78→Ala
GST-Tat_K41A/R78A_	Lys41, Arg78→Ala
GST-Tat_R78A/G79A/D80A_	Arg78, Gly79, Asp80→Ala
GST-Tat_(G48-R57)A/K(12,41)A/R78A_	amino acids 48–57, Lys12, Lys41, and Arg78→Ala

The plasmids expressing different Tat mutants were obtained by cloning a PCR-amplified fragment into the BamHI and EcoRI sites of the vector pGEX-2T. Templates for amplification were plasmid pGEX2T-Tat-derived. All the constructs were verified by DNA sequencing. Recombinant wild type HIV-1 Tat and the different Tat mutants were expressed in Escherichia coli as glutathione S-transferase (GST) fusion proteins and purified to homogeneity from bacterial lysates by glutathione-sepharose affinity chromatography (Amersham Pharmacia Biotech) according to the manufacturer's instructions. The purity and integrity of the protein product were routinely checked by SDS-polyacrylamide gel electrophoresis and 0.25% Coomassie brilliant blue R-250 staining. The purified proteins were stored in aliquots at −80°C until use[8].

### Surface plasmon resonance assay

To characterize Tat binding with heparin, kinetic properties and competitive inhibition were examined using a surface plasmon resonance assay (SPR, BIAcore X, Uppsala, Sweden). For this purpose, heparin was immobilized to CM5 sensor chip according to established method[33], [34]. The immobilization procedure was carried out at 25°C and at a constant flow rate of 5 µl/min in HBS-EP (0.01 M HEPES, pH 7.4, 0.15 M NaCl, 3 mM EDTA, and 0.005% polysorbate 20 [v/v]). To assess the real-time binding capacity, 50 µl of GST-Tat or the relevant mutant was injected over the sensor chip surface with the immobilized heparin, followed by 5 min of washing with HBS-EP buffer. The sensor chip surface was regenerated using 2 M NaCl. All binding experiments were performed at 25°C with a constant flow rate of 15 µl/min HBS-EP. To correct for nonspecific binding and bulk refractive index change, a blank channel (FC2) without heparin was employed as a control for each experiment. Sensorgrams for all binding interactions were recorded in real time and analyzed after subtraction of channel blanks. Changes in mass due to the binding response were recorded as resonance units (RU). Binding kinetics and affinities were calculated using BIAcore software 3.1. Competitive inhibition experiments were conducted with the same protocol except a 50 µl mixture of GST-Tat or the relevant mutant and inhibitors that had been preincubated for 3 min at 37°C were used.

### Internalization of GST-Tat and its mutants[10]

Three hundred microliter of SLK cell suspensions were seeded on each cover slips(3×10^5^ cells/ml in RPMI1640 with 10% FCS). After 12 h, GST-Tat or the relevant mutant protein (1.6 µg/ml) was added to the cell culture medium and incubated for 24 h. Cells were then washed three times with phosphate buffered saline (PBS) and fixed with cold 4% (v/v) paraformaldehyde for 30 min at room temperature. Subsequently, the cover slips were washed with PBS, permeabilized for 15 min in PBS containing 0.1% (v/v) Triton X-100, and blocked with 1.25% (w/v) heat-denatured BSA for 30 min at room temperature. Cells were then incubated with an anti-GST monoclonal antibody (sc-459; [1∶100]) at 4°C overnight, followed by incubation with FITC-conjugated goat anti-rabbit IgG (1∶500) at 37°C for another 30 min, and imaged using immunofluorescence confocal microscopy (LSM510-Meta, Carl Zeiss, Germany).

### Cell adhesion assay

To guarantee the same amount of tested protein was coated, anti-GST antibody was adsorbed to polystyrene microplates (Costar, Cambridge, Mass.) by overnight incubation at 4°C in 100 mM carbonate buffer (pH 9.6) at a concentration of 10 µg/ml. The plates were subsequently washed three times with tris-buffered saline+Tween 20 (TBS-T) and then filled with 1.25% BSA-PBS and incubated for 2 h at 37°C. After three washes with TBS-T, 40 µg/ml of GST alone, GST-Tat or the relevant mutant Tat protein was added to the wells and incubated for 1 hour at 37°C. The plate was next washed three times with PBS-T. Then 100 µl of SLK cell suspension was added to each well (3×10^5^ cells/ml in RPMI1640 with 10% FCS). After incubation for 1 hour at 37°C in a 5% CO2 atmosphere, non-adherent cells were removed by careful aspiration and three washes with RPMI1640. Adherent cells were fixed, stained, and quantitated as described[35]. A heparin competition cell adhesion assay was conducted with the same procedure except that heparin at defined concentration was added to each well. In the cell surface HS-blocked cell adhesion assay, cell surface HS was blocked by preincubation with function-blocking monoclonal antibody (MAB2040) against cell membrane HS at 37°C for 1 hour before being added to each well. Each experiment was performed in triplicate and replicated three times.

### Integrin clustering assay

The protein coating and cell treatment were similiar as the procedure of cell adhesion except using acid-washed cover slips instead of polystyrene microplates. The adherent cells were then fixed in 4%(v/v) paraformaldehyde for 30 min, permeabilized with 0.1%(v/v) Triton X-100 in PBS for 10 min at room temperature, and treated with blocking buffer (1.25% (w/v) BSA in PBS). Cells were then incubated with monoclonal anti-12G10 (1∶100) directed against the activated-β1 integrin at 4°C overnight, followed by incubation with Cy3-conjugated anti-mouse IgG (1∶1000) at 37°C for another 30 min and imaged using immunofluorescence confocal microscopy. A heparin-saturation integrin clustering assay was conducted using the same procedure except that heparin (final concentration 1 mg/ml) was added to the wells and incubated for 1 hour with GST, GST-Tat or the appropriate mutant Tat protein before adding cells.

## Supporting Information

Text S1 Supplementary Methods and Results
(0.03 MB DOC)

Figure S1 The representative MS-MS spectrum of GST-Tat. A, The MS-MS spectrum of GST-Tat peptide, LEPWKHPGSQPK, with Xcorr score 2.48; and B, The MS-MS spectrum of GST-Tat peptide, RPPQGSQTHQVSLSK, with Xcorr score 4.03.
(0.97 MB TIF)

Figure S2 The MS-MS spectrum of GST-Tat mutant GST-Tat(G48-R57)A. The peptide ALGISYAAAAAAAAAAPPQGSQTHQVSLSK, with Xcorr score of 5.62, indication of the 48–57 residues substituted by Alanine (The mutated amino acids are underlined).
(0.53 MB TIF)

Figure S3 The MS-MS spectrum of GST-Tat mutant GST-TatK(12,41)A/R78A. A, The MS-MS spectrum of GST-TatK(12,41)A/R78A peptide, LEPWAHPGSQPK, with Xcorr score 2.78, indication of the twelfth residue Lysine of Tat substituted by Alanine; B, The MS-MS spectrum of GST-TatK(12,41)A/R78A peptide, CCFHCQVCFITAALGISYGR, with Xcorr score 5.32, indication of the forty-first residue Lysine of Tat substituted by Alanine; and C, The MS-MS spectrum of GST-TatK(12,41)A/R78A peptide, QPTSQSAGDPTGPK, with Xcorr score 2.8, indication of the seventy-eighth Arg of Tat substituted by Alanine. The mutated amino acids are underlined.
(1.65 MB TIF)

Figure S4 The MS-MS spectrum of GST-Tat mutant GST-Tat(G48-R57)A/K(12,41)A/R78A. A, The MS-MS spectrum of GST-Tat(G48-R57)A/K(12,41)A/R78A peptide, LEPWAHPGSQPK, with Xcorr score 2.9, indication of the twelfth residue Lysine of Tat substituted by Alanine; B, The MS-MS spectrum of GST-Tat(G48-R57)A/K(12,41)A/R78A peptide, CCFHCQVCFITAALGISYAAAAAAAAAAPPQGSQTHQVSLSK, with Xcorr score 4.83, indication of the forty-first residue Lysine and the 48–57 residues of Tat substituted by Alanine; and C, The MS-MS spectrum of GST-Tat(G48-R57)A/K(12,41)A/R78A peptide, QPTSQSAGDPTGPKE, with Xcorr score 3.14, indication of the seventy-eighth Arg of Tat substituted by Alanine. The mutated amino acids are underlined.
(1.67 MB TIF)
