# Supplementary material for: A Triad of Lys12, Lys41, Arg78 Spatial Domain, a Novel Identified Heparin Binding Site on Tat Protein, Facilitates Tat-Driven Cell Adhesion
Source: PLoS One. 2008 Jul 16;3(7):e2662. doi: 10.1371/journal.pone.0002662 (PMC3278312; doi:10.1371/journal.pone.0002662)
Supplement: Text S1 — Supplementary Methods and Results (0.03 MB DOC) [file pone.0002662.s001.doc]

**Text S1. Supplementary Methods and Results**

**Methods**

*In-gel proteolysis and LC-MS analysis* The protein samples of GST-Tat, GST-Tat_(G48-R57)A_, GST-Tat_K(12,41)A/R78A_ and GST-Tat_(G48-R57)A/K(12,41)A/R78A_ were separated by 12% SDS-PAGE gel. The gel bands of interest were exercised from the Coomassie stained SDS–PAGE gel with a steel scalpel, and then were cut into small pieces and destained in an Eppendorf tube by washing sequentially with 100μl of 30% CH_3_CN/100mM ammonium bicarbonate. The washing step was repeated until the gel bands were clear. Then they were reduced by DTT and the free Cysteines were modified by IAA. Digestion reaction was carried out with sequencing grade-modified trypsin (Promega, about 20:1) in 40 mM NH_4_HCO_3_/ 10% CH_3_CN buffer at 37°C for 16 hours. Peptides were recovered by extraction with 60% CH_3_CN/0.5% TFA buffer, dried in vacuum and resuspended with buffer containing 0.5% formic acid. LC-MS/MS analysis was performed using LTQ mass spectrometer (Thermo, San Jose, CA, USA) equipped with a MS pump and an autosampler. The HPLC solvents used were 0.1% formic acid in water (v/v, buffer A) and 0.1% formic acid in 100% acetonitrile (v/v, buffer B). The reversed-phase C18 column with 0.18-mm id and 100-mm length was from Thermo. The mass spectrometer was set to one full MS scan followed by six MS/MS scans on the six most intense ions with the application of Dynamic Exclusion. Data collection was conducted by Xcalibur software version 1.4 (Thermo).

The acquired MS/MS spectra were automatically searched against the relevant protein sequence using the TurboSEQUEST program in the BioWorks 3.1 software suite (Thermo). The spectra corresponding to the mutant peptides were manually checked.

**Results**

Results indicated that all the data released confirmed the sequences of being mutated (Supplementary Figure S1, S2, S3, S4).

**FIGURE LEGENDS**

**Figure S1.** **The representative MS-MS spectrum of GST-Tat.** **A,** The MS-MS spectrum of GST-Tat peptide, LEPWKHPGSQPK, with Xcorr score 2.48; and **B,** The MS-MS spectrum of GST-Tat peptide, RPPQGSQTHQVSLSK, with Xcorr score 4.03.

**Figure S2.** **The MS-MS spectrum of GST-Tat mutant GST-Tat_(G48-R57)A_.** The peptide ALGISYAAAAAAAAAAPPQGSQTHQVSLSK, with Xcorr score of 5.62, indication of the 48-57 residues substituted by Alanine (The mutated amino acids are underlined).

**Figure S3.** **The MS-MS spectrum of GST-Tat mutant GST-Tat_K(12,41)A/R78A_.** **A,** The MS-MS spectrum of GST-Tat_K(12,41)A/R78A_ peptide, LEPWAHPGSQPK, with Xcorr score 2.78, indication of the twelfth residue Lysine of Tat substituted by Alanine; **B,** The MS-MS spectrum of GST-Tat_K(12,41)A/R78A_ peptide, CCFHCQVCFITAALGISYGR, with Xcorr score 5.32, indication of the forty-first residue Lysine of Tat substituted by Alanine; and **C,** The MS-MS spectrum of GST-Tat_K(12,41)A/R78A_ peptide, QPTSQSAGDPTGPK, with Xcorr score 2.8, indication of the seventy-eighth Arg of Tat substituted by Alanine. The mutated amino acids are underlined.

**Figure S4.** **The MS-MS spectrum of GST-Tat mutant GST-Tat_(G48-R57)A/K(12,41)A/R78A_.** **A,** The MS-MS spectrum of GST-Tat_(G48-R57)A/K(12,41)A/R78A_ peptide, LEPWAHPGSQPK, with Xcorr score 2.9, indication of the twelfth residue Lysine of Tat substituted by Alanine; **B,** The MS-MS spectrum of GST-Tat_(G48-R57)A/K(12,41)A/R78A_ peptide, CCFHCQVCFITAALGISYAAAAAAAAAAPPQGSQTHQVS LSK, with Xcorr score 4.83, indication of the forty-first residue Lysine and the 48-57 residues of Tat substituted by Alanine; and **C,** The MS-MS spectrum of GST-Tat_(G48-R57)A/K(12,41)A/R78A_ peptide, QPTSQSAGDPTGPKE, with Xcorr score 3.14, indication of the seventy-eighth Arg of Tat substituted by Alanine. The mutated amino acids are underlined.
