# Supplementary figures and images for: A Triad of Lys12, Lys41, Arg78 Spatial Domain, a Novel Identified Heparin Binding Site on Tat Protein, Facilitates Tat-Driven Cell Adhesion
Source: PLoS One. 2008 Jul 16;3(7):e2662. doi: 10.1371/journal.pone.0002662 (PMC3278312; doi:10.1371/journal.pone.0002662)

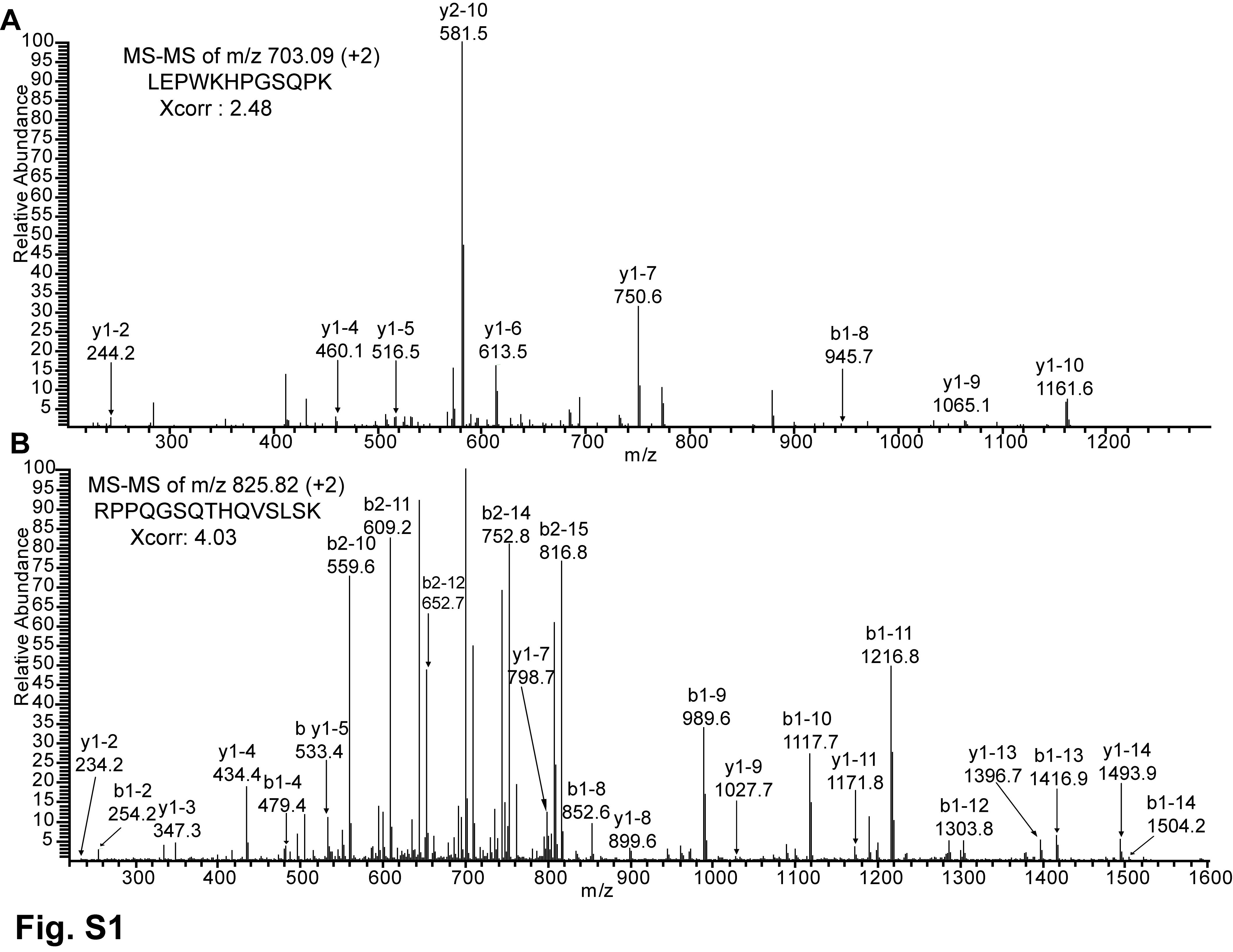

Supplement: Figure S1 — The representative MS-MS spectrum of GST-Tat. A, The MS-MS spectrum of GST-Tat peptide, LEPWKHPGSQPK, with Xcorr score 2.48; and B, The MS-MS spectrum of GST-Tat peptide, RPPQGSQTHQVSLSK, with Xcorr score 4.03. (0.97 MB TIF) [file pone.0002662.s002.tif]

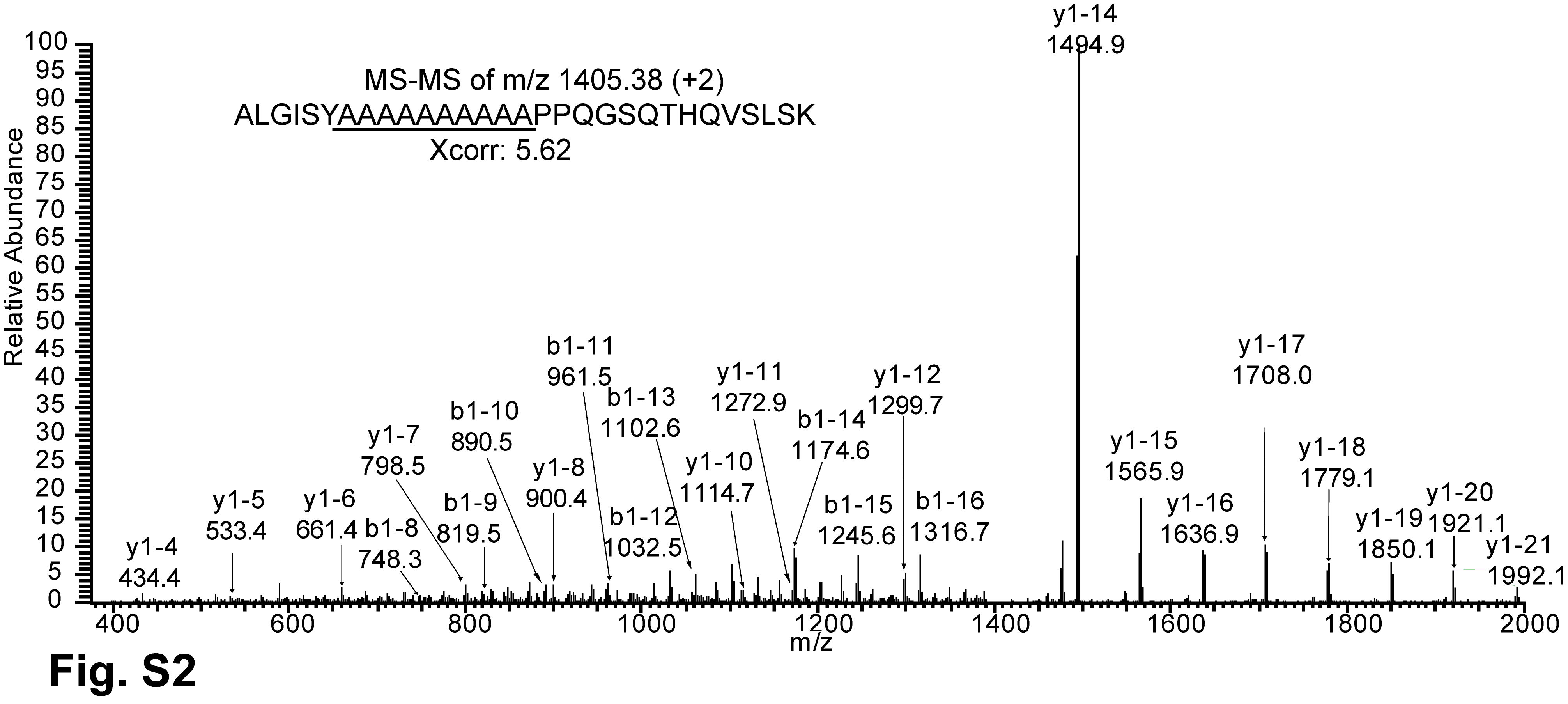

Supplement: Figure S2 — The MS-MS spectrum of GST-Tat mutant GST-Tat(G48-R57)A. The peptide ALGISYAAAAAAAAAAPPQGSQTHQVSLSK, with Xcorr score of 5.62, indication of the 48–57 residues substituted by Alanine (The mutated amino acids are underlined). (0.53 MB TIF) [file pone.0002662.s003.tif]

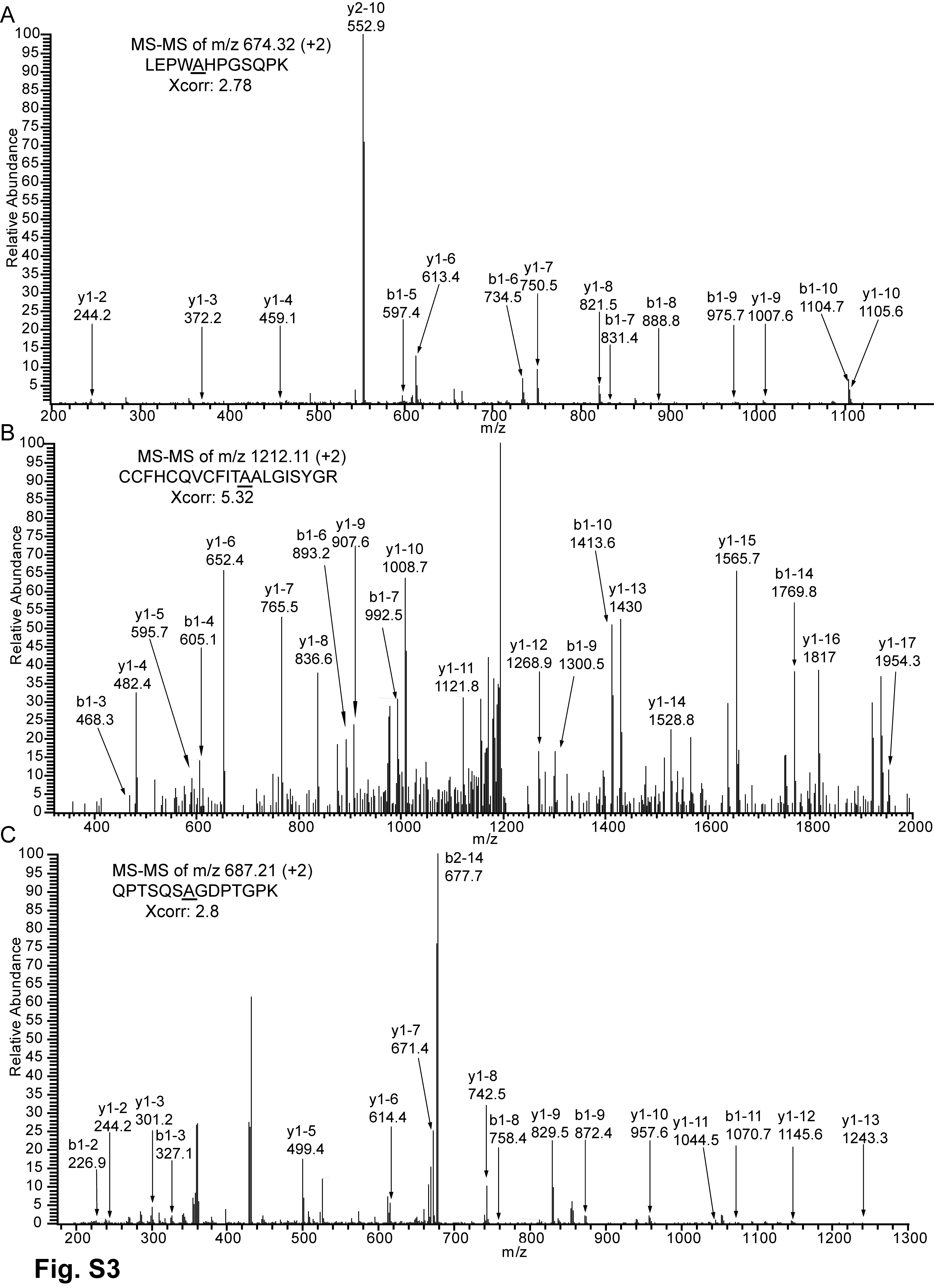

Supplement: Figure S3 — The MS-MS spectrum of GST-Tat mutant GST-TatK(12,41)A/R78A. A, The MS-MS spectrum of GST-TatK(12,41)A/R78A peptide, LEPWAHPGSQPK, with Xcorr score 2.78, indication of the twelfth residue Lysine of Tat substituted by Alanine; B, The MS-MS spectrum of GST-TatK(12,41)A/R78A peptide, CCFHCQVCFITAALGISYGR, with Xcorr score 5.32, indication of the forty-first residue Lysine of Tat substituted by Alanine; and C, The MS-MS spectrum of GST-TatK(12,41)A/R78A peptide, QPTSQSAGDPTGPK, with Xcorr score 2.8, indication of the seventy-eighth Arg of Tat substituted by Alanine. The mutated amino acids are underlined. (1.65 MB TIF) [file pone.0002662.s004.tif]

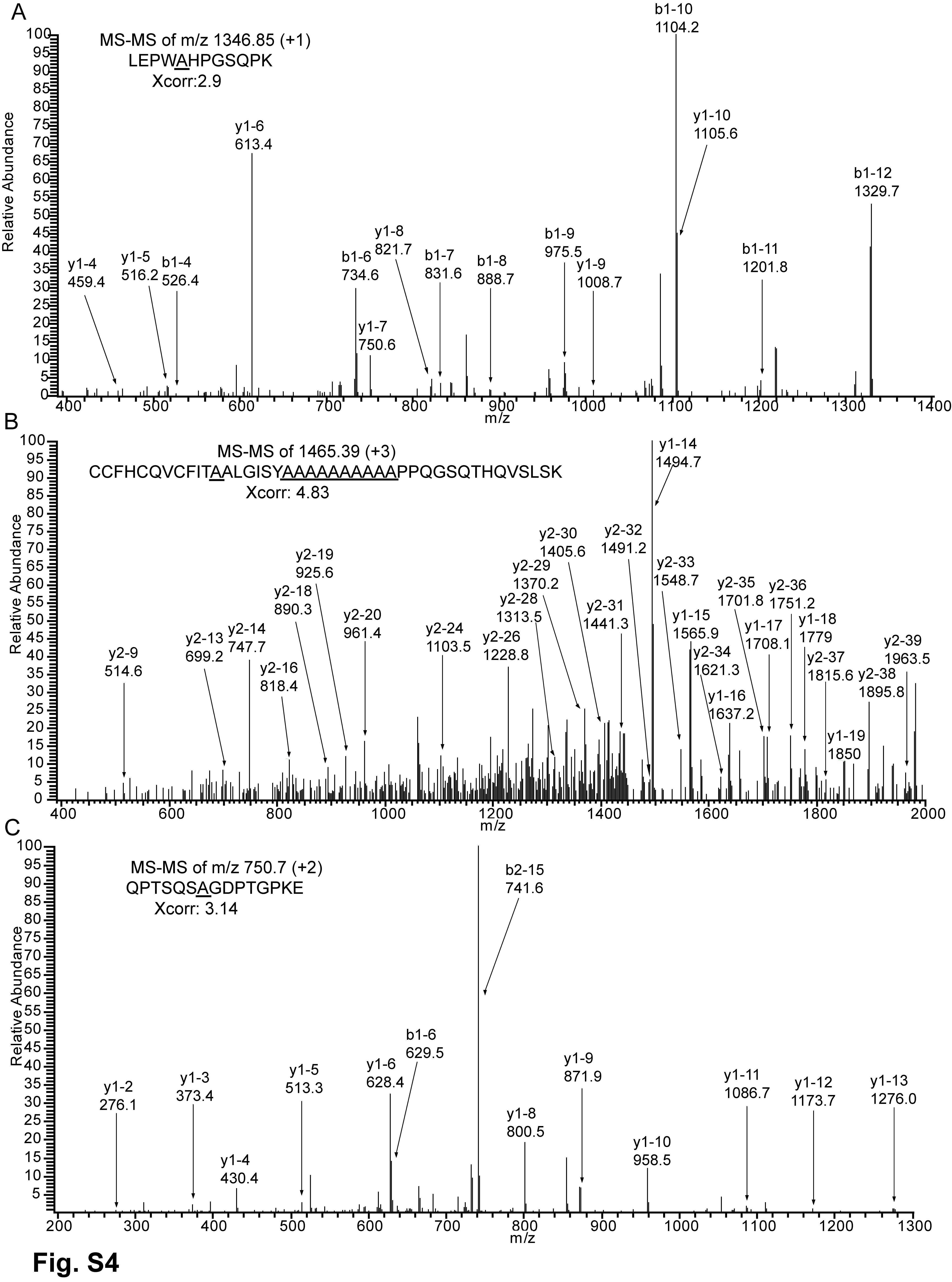

Supplement: Figure S4 — The MS-MS spectrum of GST-Tat mutant GST-Tat(G48-R57)A/K(12,41)A/R78A. A, The MS-MS spectrum of GST-Tat(G48-R57)A/K(12,41)A/R78A peptide, LEPWAHPGSQPK, with Xcorr score 2.9, indication of the twelfth residue Lysine of Tat substituted by Alanine; B, The MS-MS spectrum of GST-Tat(G48-R57)A/K(12,41)A/R78A peptide, CCFHCQVCFITAALGISYAAAAAAAAAAPPQGSQTHQVSLSK, with Xcorr score 4.83, indication of the forty-first residue Lysine and the 48–57 residues of Tat substituted by Alanine; and C, The MS-MS spectrum of GST-Tat(G48-R57)A/K(12,41)A/R78A peptide, QPTSQSAGDPTGPKE, with Xcorr score 3.14, indication of the seventy-eighth Arg of Tat substituted by Alanine. The mutated amino acids are underlined. (1.67 MB TIF) [file pone.0002662.s005.tif]
